# Succinate dehydrogenase expression in breast cancer

**DOI:** 10.1186/2193-1801-2-299

**Published:** 2013-07-03

**Authors:** Sewha Kim, Do Hee Kim, Woo-Hee Jung, Ja Seung Koo

**Affiliations:** Department of Pathology, Yonsei University College of Medicine, Seoul, South Korea

**Keywords:** Breast, Metabolism, Mitochondria, Succinate dehydrogenase

## Abstract

The aim of this study was to investigate succinate dehydrogenase (SDH) expression in breast cancer according to breast cancer molecular subtype using immunohistochemistry and to assess the clinical implications of SDH expression. Immunohistochemical staining for ER, PR, HER-2, Ki-67, HIF-1α, SDHA, and SDHB was performed on tissue microarrays of 721 breast cancers. According to the immunohistochemistry results for ER, PR, HER-2, and Ki-67 and fluorescence in situ hybridization (FISH) results for HER-2, breast cancer molecular subtypes were classified into luminal A, luminal B, HER-2, and triple-negative breast cancer (TNBC). HER-2 subtype breast cancers most frequently showed high-level expression of SDHA in tumor cells, while the luminal A subtype most frequently showed low or negative expression of SDHA in tumor cells (*P* = 0.032). Stromal SDHB expression rate was highest in HER-2 subtype and lowest in TNBC (*P* < 0.001). SDHA-negative breast cancers were associated with younger age at diagnosis (*P* = 0.012), and SDHB-negative breast cancers with lower histologic grade (*P* = 0.044) and lower Ki-67 labeling index (LI) (*P* = 0.046). Tumor phenotypes according to the SDH status were SDHA(+)/SDHB(+) > SDHA(–)/SDHB(–) > SDHA(–)/SDHB(+) > SDHA(+)/SDHB(–) in order of frequency. The stromal phenotypes were SDHA(–)/SDHB(–) > SDHA(+)/SDHB(+) > SDHA(–)/SDHB(+) > SDHA(+)/SDHB(–). In conclusion*,* loss of SDHA or SDHB expression was observed in about 3% of breast cancers in this study. Low SDH expression status in breast tumor cells was associated with younger age at diagnosis and low-grade histology.

**Electronic supplementary material:**

The online version of this article (doi:10.1186/2193-1801-2-299) contains supplementary material, which is available to authorized users.

## Introduction

Succinate dehydrogenase (SDH) (succinate-coenzyme Q reductase, respiratory Complex II) catalyzes the oxidation of succinate to fumarate with the reduction of ubiquinone to ubiquinol. Through the coupling of these two reactions in the inner mitochondrial membrane, SDH links glucose oxidation in the TCA cycle with ATP production in the mitochondria (Cardaci & Ciriolo 2012;King et al. 2006;Barletta & Hornick 2012;Van Nederveen et al. 2009). SDH is composed of four subunits; SDHA, SDHB, SDHC and SDHD. The first two are hydrophilic proteins protruding into the mitochondrial matrix, and the second two are hydrophobic proteins that anchor the catalytic core into the inner mitochondrial membrane (Cardaci & Ciriolo 2012; King et al. 2006; Barletta & Hornick 2012; Van Nederveen et al. 2009).

Germline loss-of-function SDH mutations are associated with several tumors such as pheochromocytoma and paraganglioma, gastrointestinal stromal tumor (GIST), and renal cell carcinoma (Hensen & Bayley 2011; Kantorovich et al. 2010; Anderson et al. 2004; Astuti et al. 2001; Miettinen et al. 2011; Badve et al. 2011; Ricketts et al. 2008). Among these, GIST and renal cell carcinoma are reported to display histologic features that distinguish SDH-deficient tumors (Miettinen et al. 2011). Although SDH germline mutation may be accurately evaluated by gene sequencing, a few recent studies have shown very high sensitivity for immunohistochemical detection of SDH mutation in GIST, pheochromocytoma, and paraganglioma (Van Nederveen et al. 2009; Gill et al. 2010a; Miettinen et al. 2013; Janeway et al. 2011).

To date, few studies have investigated SDH expression in breast cancer. We investigated SDH expression status according to breast cancer molecular subtype using immunohistochemical methods and assessed the clinical implications of SDH expression in breast cancer.

## Materials and methods

### Patient selection

Patients who were diagnosed with invasive ductal carcinoma, not otherwise specified and underwent surgical excision at Severance Hospital between January 2002 and December 2005 were included in the study. Patients who received preoperative hormonal therapy or neoadjuvant chemotherapy were excluded. All hematoxylin and eosin (H&E)-stained slides for each case were retrospectively reviewed by breast pathologists (Koo JS). The histological grade was assessed using the Nottingham grading system.(Elston & Ellis 1991) The clinicopathologic parameters evaluated for each breast cancer included patient age at initial diagnosis, lymph node metastasis, tumor recurrence, distant metastasis, and patient survival. The Institutional Review Board of Yonsei University Severance Hospital approved this study.

### Tissue microarray

On H&E-stained slides of tumors, a representative area was selected and a corresponding spot was marked on the surface of the paraffin block. Using a biopsy needle, the selected area was punched out and a 3-mm core of tumor tissue was placed onto a 6 × 5 recipient block. Two tissue cores were extracted from each sample to minimize bias. Each tissue core was assigned a unique microarray location number that was linked to clinicopathologic data in a database.

### Immunohistochemistry

All immunohistochemical staining was performed on formalin-fixed, paraffin-embedded tissue sections using antibodies as indicated (Table 1). Briefly, 5-μm-thick sections were obtained with a microtome, transferred onto adhesive slides, and dried at 62 °C for 30 min. After incubation with primary antibodies, immunodetection was performed with biotinylated antimouse immunoglobulin, followed by peroxidase-labeled streptavidin using a labeled streptavidin biotin kit with 3,3′-diaminobenzidine chromogen as substrate. The primary antibody incubation step was omitted in the negative control. Slides were counterstained with Harris hematoxylin.

**Table 1:** **Clone, dilution, and source of antibodies used**

Antibody	Clone	Dilution	Company
HIF-1α	EP1215Y	1:100	Biocare, Yorba Linda, CA, USA
SDHA	2E3GC12FB2AE2	1:100	Abcam, Cambridge, UK
SDHB	21A11AE7	1:100	Abcam, Cambridge, UK
Tumor phenotype-related			
ER	SP1	1:100	Thermo Scientific, CA, USA
PR	PgR	1:50	DAKO, Denmark
HER-2	Polyclonal	1:1500	DAKO, Denmark
Ki-67	MIB-1	1:150	DAKO, Glostrup, Denmark

*ER* estrogen receptor; *PR* progesterone receptor, *EGFR* epidermal growth factor receptor.

### Interpretation of immunohistochemical staining

All immunohistochemical markers were assessed by light microscopy. Parameters such as ER, PR, and HER-2 status were obtained from patients’ pathologic reports. A cut-off value of 1% or more positively stained nuclei was used to define ER and PR positivity (Hammond et al. 2010). HER-2 staining was analyzed according to the American Society of Clinical Oncology (ASCO)/College of American Pathologists (CAP) guidelines using the following categories: 0, no immunostaining; 1+, weak incomplete membranous staining, less than 10% of tumor cells; 2+, complete membranous staining, either uniform or weak in at least 10% of tumor cells; and 3+, uniform intense membranous staining in at least 30% of tumor cells (Wolff AC, et al. 2007). HER-2 immunostaining was recorded as positive when strong (3+) membranous staining was observed, whereas staining categories 0 to 1+ were recorded as negative. Samples showing 2+ HER-2 expression were evaluated for HER-2 amplification by fluorescence in situ hybridization (FISH).

**Figure 1:**
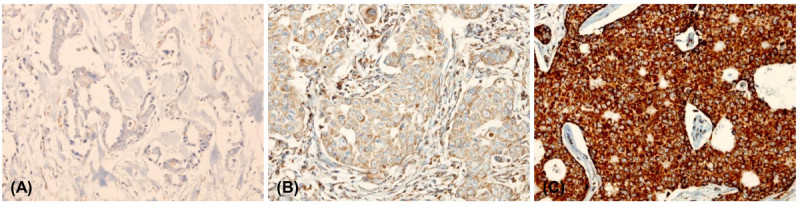
**Immunohistochemical evaluation of SDHB expression. (A) negative (B) low positive (C) high positive.**

Immunohistochemical staining for SDHA and SDHB showed cytoplasmic expression with a granular pattern. Complete loss of expression was considered negative, while cytoplasmic expression in less than 50% of tumor cells was considered low expression, and cytoplasmic expression in more than 50% of tumor cells was considered high expression (Figure 1). HIF-1α was considered positive when more than 10% of tumor cells showed strong cytoplasmic or nuclear expression.

### Fish analysis

FISH was performed using a PathVysion HER-2 DNA Probe Kit (Vysis, Downers Grove, IL, USA) according to the manufacturer’s instructions. Invasive tumors were first examined on hematoxylin-eosin-stained slides to confirm histology. HER-2 gene copy number on the slides was evaluated using an epifluorescence microscope (Olympus, Tokyo, Japan) according to the ASCO/CAP guidelines (Wolff AC, et al. 2007). At least 60 tumor cell nuclei in three separate regions were investigated for HER-2 and chromosome 17 signals. An absolute HER-2 gene copy number lower than 4 or an HER-2 gene/chromosome 17 copy number ratio (HER-2/Chr17 ratio) less than 1.8 was considered HER-2-negative. An absolute HER-2 copy number between 4 and 6 or an HER-2/Chr17 ratio between 1.8 and 2.2 was considered HER-2-equivocal. An absolute HER-2 copy number greater than 6 or an HER-2/Chr17 ratio higher than 2.2 was considered HER-2-positive.

### Tumor phenotype classification

Breast cancer phenotypes were classified according to the immunohistochemistry results for ER, PR, HER-2, and Ki-67 and the FISH results for HER-2 as follows (Goldhirsch A, et al. 2011): Luminal A subtype as ER- or/and PR-positive and HER-2-negative and Ki-67 LI <14%; Luminal B subtype as ER- or/and PR-positive and HER-2-negative and Ki-67 LI ≥14%; HER-2 positive as ER- or/and PR-positive and HER-2 overexpressed or/and amplified; HER-2-overexpression subtype as ER- and PR-negative and HER-2 overexpressed or/and amplified; and triple-negative breast cancer (TNBC) subtype as ER-, PR-, and HER-2-negative.

### Statistical analysis

Data were analyzed using SPSS for Windows, Version 12.0 (SPSS Inc., Chicago, IL, USA). Student’s t and Fisher’s exact tests were used to evaluate continuous and categorical variables, respectively. The significance level was set at 0.05. The time to tumor recurrence and overall survival were evaluated by Kaplan-Meier and log-rank statistics. Multivariate regression analysis was performed using the Cox proportional hazards model.

## Results

### Patients’ characteristics

Clinicopathological characteristics according to breast cancer molecular subtype are shown in Table 2. Molecular subtypes among these 721 breast cancers included 302 luminal A (41.9%), 168 luminal B (23.3%), 69 HER-2 type (9.6%), and 182 TNBC (25.2%). TNBC showed higher histologic grade (*P* < 0.001), higher T stage (*P* = 0.007), and higher Ki-67 LI (*P* < 0.001) than other molecular subtypes. The HER-2 subtype was positively associated with age (*P* = 0.005) and with tumor recurrence and death (*P* = 0.001).

**Table 2:** **Clinicopathologic characteristics according to breast cancer phenotype**

Parameters	Total ***n*** = 721 (%)	Luminal A ***n*** = 302 (%)	Luminal B ***n*** = 168 (%)	HER-2 ***n*** = 69 (%)	TNBC ***n*** = 182 (%)	***P***-value
		***n*** = 302 (%)	***n*** = 168 (%)	***n*** = 69 (%)	***n*** = 182 (%)	
Age (years, mean ± SD)	49.6 ± 10.9	50.6 ± 10.4	48.4 ± 10.0	52.4 ± 10.0	48.0 ± 12.4	0.005
Histologic grade						<0.001
I	120 (16.6)	92 (30.5)	20 (11.9)	1 (1.4)	7 (3.8)	
II	364 (50.5)	182 (60.3)	91 (54.2)	36 (52.2)	55 (30.2)	
III	237 (32.9)	28 (9.3)	57 (33.9)	32 (46.4)	120 (65.9)	
Tumor stage						0.007
T1	350 (48.5)	167 (55.3)	85 (50.6)	30 (43.5)	68 (37.4)	
T2	356 (49.4)	127 (42.1)	81 (48.2)	38 (55.1)	110 (60.4)	
T3	15 (2.1)	8 (2.6)	2 (1.2)	1 (1.4)	4 (2.2)	
Nodal stage						0.060
N0	425 (58.9)	171 (56.6)	92 (54.8)	42 (60.9)	120 (65.9)	
N1	192 (26.6)	89 (29.5)	43 (25.6)	13 (18.8)	47 (25.8)	
N2	65 (9.0)	27 (8.9)	18 (10.7)	9 (13.0)	11 (6.0)	
N3	39 (5.4)	15 (5.0)	15 (8.9)	5 (7.2)	4 (2.2)	
Estrogen receptor status						<0.001
Negative	261 (36.2)	5 (1.7)	5 (3.0)	69 (100.0)	182 (0.0)	
Positive	460 (63.8)	297 (98.3)	163 (97.0)	0 (0.0)	0 (0.0)	
Progesterone receptor status						<0.001
Negative	346 (48.0)	49 (16.2)	46 (27.4)	69 (100.0)	182 (100.0)	
Positive	375 (52.0)	253 (83.8)	122 (72.6)	0 (0.0)	0 (0.0)	
HER-2 status						<0.001
Negative	573 (79.5)	302 (100.0)	89 (53.0)	0 (0.0)	182 (100.0)	
Positive	148 (20.5)	0 (0.0)	79 (47.0)	69 (100.0)	0 (0.0)	
Ki-67 LI (%, mean ± SD)	17.3 ± 18.4	4.7 ± 3.7	19.6 ± 12.6	19.3 ± 12.8	35.1 ± 23.0	<0.001
Tumor recurrence	63 (8.7)	14 (4.6)	13 (7.7)	11 (15.9)	25 (13.7)	0.001
Patient death	60 (8.3)	12 (4.0)	13 (7.7)	11 (15.9)	24 (13.2)	<0.001
Duration of clinical follow-up (months, mean ± SD)	70.0 ± 31.2	72.4 ± 29.3	70.2 ± 30.0	65.2 ± 34.3	67.8 ± 34.2	0.234

*TNBC* triple negative breast cancer.

### HIF-1α and sdh status according to molecular subtype of breast cancer

**Figure 2:**
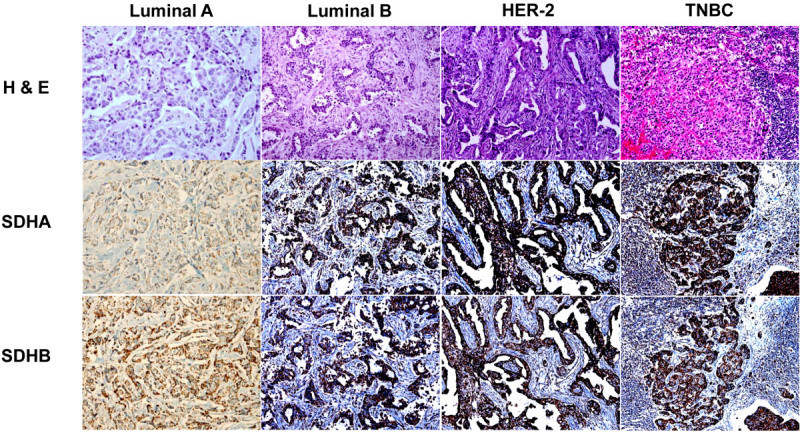
**Expression of SDHA and SDHB according to the molecular subtype of breast cancer.** Tumor cells showed highest SDHA expression in the HER-2 subtype and lowest SDHA expression in the luminal A subtype. Stromal elements expressed SDHA at lower levels than tumor cells.

**Figure 3:**
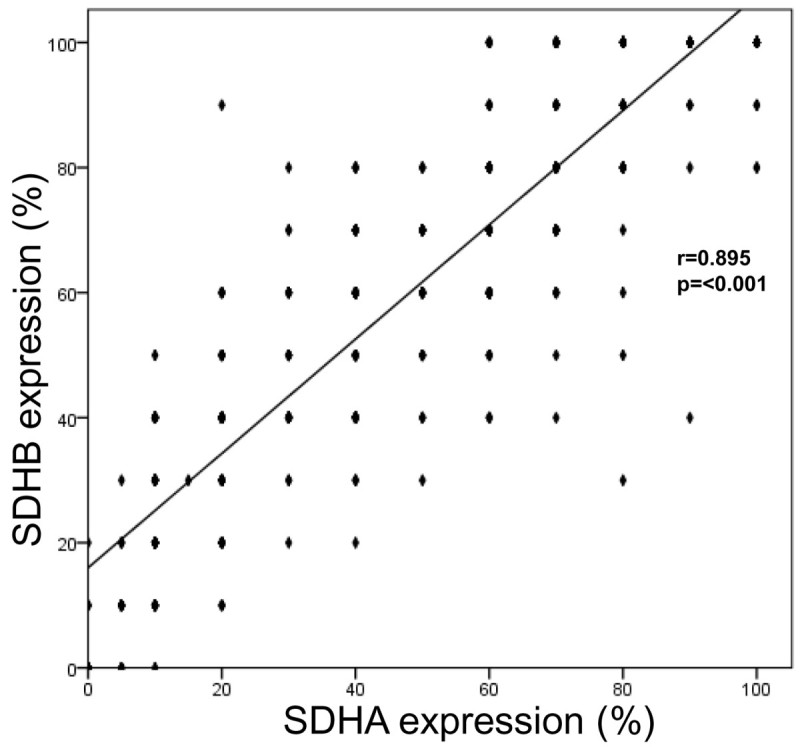
**Correlation between SDHA and SDHB expression.**

HIF-1α, SDHA, and SDHB expression according to breast cancer subtype are shown in Table 3 and Figure 2. Only 24 (3.3%) and 4 (0.6%) of 721 breast cancers displayed HIF-1α expression in the nucleus and cytoplasm, respectively. The HER-2 tumor subtype showed the highest frequency of high SDHA expression, and the luminal A subtype most frequently showed low or negative SDHA expression (*P* = 0.032). Stromal SDHB expression frequency was highest in the HER2 subtype and lowest in TNBC (*P* < 0.001). Stromal SDHA expression was detected most frequently in the HER-2 subtype, although the correlation was not significant (*P* = 0.063). Nuclear HIF-1α expression and SDHA/SDHB expression did not correlate significantly (Table 4); however, SDHA and SDHB expression were correlated significantly (r = 0.895, *P* < 0.001, Figure 3).

**Table 3:** **Expression of HIF-1α and SDH according to tumor phenotype**

Parameters	Total ***n*** = 721 (%)	Luminal A ***n*** = 302 (%)	Luminal B ***n*** = 168 (%)	HER-2 ***n*** = 69 (%)	TNBC ***n*** = 182 (%)	P-value
Nuclear HIF-1α						0.319
Negative	697 (96.7)	296 (98.0)	161 (95.8)	65 (94.2)	175 (96.2)	
Positive	24 (3.3)	6 (2.0)	7 (4.2)	4 (5.8)	7 (3.8)	
Cytoplasmic HIF-1α						0.596
Negative	717 (99.4)	301 (99.7)	166 (98.8)	69 (100.0)	181 (99.5)	
Positive	4 (0.6)	1 (0.3)	2 (1.2)	0 (0.0)	1 (0.5)	
Tumoral SDHA						**0.032**
Negative/Low	41/319 (49.9)	21/143 (54.3)	8/76 (50.0)	5/19 (34.8)	7/81 (48.4)	
High	361 (50.1)	138 (45.7)	84 (50.0)	45 (65.2)	94 (51.6)	
Stromal SDHA						0.063
Negative	665 (92.2)	286 (94.7)	153 (91.1)	59 (85.5)	167 (91.8)	
Positive	56 (7.8)	16 (5.3)	15 (8.9)	10 (14.5)	15 (8.2)	
Tumoral SDHB						0.291
Negative/Low	24/245 (37.3)	15/103 (39.1)	3/56 (35.1)	3/19 (31.9)	3/67 (38.5)	
High	452 (62.7)	184 (60.9)	109 (64.9)	47 (68.1)	112 (61.5)	
Stromal SDHB						**<0.001**
Negative	641 (88.9)	277 (91.7)	142 (84.5)	51 (73.9)	171 (94.0)	
Positive	80 (11.1)	25 (8.3)	26 (15.5)	18 (26.1)	11 (6.0)	

*TNBC* triple negative breast cancer.

**Table 4:** **Expression of SDHA and SDHB according to HIF-1α expression status**

Factors	Nuclear HIF-1α			Cytoplasmic HIF-1α		
	Negative ***n*** = 697 (%)	Positive ***n*** = 24 (%)	***P***-value	Negative ***n*** = 717 (%)	Positive ***n*** = 4 (%)	***P***-value
Tumoral SDHA			0.247			0.518
Negative	40 (5.7)	1 (4.2)		40 (5.6)	1 (25.0)	
Low	311 (44.6)	8 (33.3)		318 (44.4)	1 (25.0)	
High	346 (49.6)	15 (62.5)		359 (50.1)	50.0)	
Stromal SDHA			0.423			1.000
Negative	644 (92.4)	21 (87.5)		661 (92.2)	4 (100.0)	
Positive	53 (7.6)	3 (12.5)		56 (7.8)	0 (0.0)	
Tumoral SDHB			0.926			**0.032**
Negative	24 (3.4)	0 (0.0)		23 (3.2)	1 (25.0)	
Low	235 (33.7)	10 (41.7)		243 (33.9)	2 (50.0)	
High	438 (62.8)	14 (58.3)		451 (62.9)	1 (25.0)	
Stromal SDHB			0.328			1.000
Negative	621 (89.1)	20 (83.3)		637 (88.8)	4 (100.0)	
Positive	76 (10.9)	4 (16.7)		80 (11.2)	0 (0.0)	

*SDH* succinate dehydrogenase.

### Clinicopathologic characteristics of breast cancer with SDHA and/or SDHB negativity

Clinicopathologic characteristics of breast cancers were compared between the SDHA/SDHB positive and negative breast cancers (Table 5). Tumor negativity for SDHA correlated with earlier age at diagnosis of breast cancer (*P* = 0.012) and with lower histologic grade (*P* = 0.062). SDHB-negative breast cancer correlated with lower histologic grade (*P* = 0.044) and lower Ki-67 LI (*P* = 0.046). There was no correlation between the expression status of SDH and that of nuclear HIF-1α (Table 4).

**Table 5:** **Clinicopathologic characteristics of breast cancer with SDHA and/or SDHB negativity in tumor cells**

Parameters	SDHA			SDHB		
	Negative ***n*** = 41 (%)	Positive ***n*** = 680 (%)	***P***-value	Negative ***n*** = 24 (%)	Positive ***n*** = 697 (%)	***P***-value
Age (years, mean ± SD)	45.4 ± 10.3	49.9 ± 10.9	**0.012**	46.9 ± 9.1	49.7 ± 10.9	0.212
Histologic grade			**0.062**			**0.044**
I/II	33 (80.5)	451 (66.3)		21 (87.5)	463 (66.4)	
III	8 (19.5)	229 (33.7)		3 (12.5)	234 (33.6)	
ER			0.405			0.286
Negative	12 (29.3)	249 (36.6)		6 (25.0)	255 (36.6)	
Positive	29 (70.7)	431 (63.4)		18 (75.0)	442 (63.4)	
PR			0.748			1.000
Negative	21 (51.2)	325 (47.8)		11 (45.8)	335 (48.1)	
Positive	20 (48.8)	355 (52.2)		13 (54.2)	362 (51.9)	
HER-2			0.693			0.800
Negative	34 (82.9)	539 (79.3)		20 (83.3)	553 (79.3)	
Positive	7 (17.1)	141 (20.7)		4 (16.7)	144 (20.7)	
Tumor stage			0.750			0.213
T1	21 (51.2)	329 (48.4)		15 (62.5)	335 (48.1)	
T2/T3	20 (48.8)	351 (51.6)		9 (37.5)	362 (51.9)	
Nodal stage			0.625			0.529
N0	26 (63.4)	399 (58.7)		16 (66.7)	409 (58.7)	
N1/N2/N3	15 (36.6)	281 (41.3)		8 (33.3)	288 (41.3)	
Molecular subtypes			0.452			0.134
Luminal A	21 (51.2)	281 (41.3)		15 (62.5)	287 (41.2)	
Luminal B	8 (19.5)	160 (23.5)		3 (12.5)	165 (23.7)	
HER-2	5 (12.2)	64 (9.4)		3 (12.5)	66 (9.5)	
TNBC	7 (17.1)	175 (25.7)		3 (12.5)	179 (25.7)	
Ki-67 LI (%, mean ± SD)	13.0 ± 12.3	17.5 ± 18.7	0.127	9.9 ± 10.0	17.5 ± 18.6	**0.046**
Tumor recurrence	4 (9.8)	59 (8.7)	0.775	1 (4.2)	62 (8.9)	0.714
Patient death	4 (9.8)	56 (8.2)	0.768	1 (4.2)	59 (8.5)	0.713

*SDH* succinate dehydrogenase, *TNBC* triple negative breast cancer.

### Correlations between clinicopathologic parameters and expression of Hif-1α and SDH

Correlation analysis between HIF-1α and SDH expression and clinicopathologic factors revealed an association of nuclear HIF-1α expression with PR negativity (*P* = 0.011), and of low or negative SDHA expression in the tumor with ER positivity (*P* = 0.044), HER-2 negativity (*P* = 0.021), and higher T stage (*P* = 0.031). Low or negative SDHB expression in the tumor was associated with higher T stage (*P* = 0.011). Stromal SDHB negativity was associated with HER-2 negativity (*P* < 0.001, Table 6).

**Table 6:** **Clinicopathologic characteristics breast cancer by expression of HIF-1α and SDH**

Parameters	Nuclear HIF-1α			Cytoplasmic HIF-1α			Tumor SDHA			Stromal SDHA			Tumor SDHB			Stromal SDHB		
	-	+	***P***	-	+	***P***	-*	+	***P***	-	+	***P***	-*	+	***P***	-	+	***P***
Histologic grade			0.270			0.601			0.342			0.105			0.870			0.528
I/II	465 (66.7)	19 (79.2)		482 (67.2)	2 (50.0)		248 (68.9)	236 (65.4)		452 (68.0)	32 (57.1)		182 (67.7)	302 (66.8)		433 (67.6)	51 (63.8)	
III	232 (33.3)	5 (20.8)		235 (32.8)	2 (50.0)		112 (31.2)	125 (34.6)		213 (32.0)	24 (42.9)		87 (32.3)	150 (33.2)		208 (32.4)	29 (36.3)	
ER			0.388			1.000			**0.044**			0.111			0.873			0.806
Negative	250 (35.9)	11 (45.8)		260 (36.3)	1 (25.0)		117 (32.5)	144 (39.9)		235 (35.3)	26 (46.4)		96 (35.7)	165 (36.5)		231 (36.0)	30 (37.5)	
Positive	447 (64.1)	13 (54.2)		457 (63.7)	3 (75.0)		243 (67.5)	217 (60.1)		430 (64.7)	30 (53.6)		173 (64.3)	287 (63.5)		410 (64.0)	50 (62.5)	
PR			**0.011**			1.000			0.118			0.165			0.645			1.000
Negative	328 (47.1)	18 (75.0)		344 (48.0)	2 (50.0)		162 (45.0)	184 (51.0)		314 (47.2)	32 (57.1)		126 (46.8)	220 (48.7)		308 (48.0)	38 (47.5)	
Positive	369 (52.9)	6 (25.0)		373 (52.0)	2 (50.0)		198 (55.0)	177 (49.0)		351 (52.8)	24 (42.9)		143 (53.2)	232 (51.3)		333 (52.0)	42 (52.5)	
HER-2			0.304			1.000			**0.021**			0.083			0.057			**<0.001**
Negative	556 (79.8)	17 (70.8)		570 (79.5)	3 (75.0)		299 (83.1)	274 (75.9)		534 (80.3)	39 (69.6)		224 (83.3)	349 (77.2)		526 (82.1)	47 (58.8)	
Positive	141 (20.2)	7 (29.2)		147 (20.5)	1 (25.0)		61 (16.9)	87 (24.1)		131 (19.7)	17 (30.4)		45 (16.7)	103 (22.8)		115 (17.9)	33 (41.3)	
Tumor stage			0.304			1.000			**0.031**			1.000			**0.011**			0.237
T1	341 (48.9)	9 (37.5)		348 (48.5)	2 (50.0)		160 (44.4)	190 (52.6)		323 (48.6)	27 (48.2)		114 (42.4)	236 (52.2)		306 (47.7)	44 (55.0)	
T2/T3	356 (51.1)	15 (62.5)		369 (51.5)	2 (50.0)		200 (55.6)	171 (47.4)		342 (51.4)	29 (51.8)		155 (57.6)	216 (47.8)		335 (52.3)	36 (45.0)	
Nodal stage			0.208			0.648			0.198			0.575			0.639			0.149
N0	414 (59.4)	11 (45.8)		422 (58.9)	3 (75.0)		221 (61.4)	204 (56.5)		394 (59.2)	31 (55.4)		162 (60.2)	263 (58.2)		384 (59.9)	41 (51.3)	
N1/N2/N3	283 (40.6)	13 (54.2)		295 (41.1)	1 (25.0)		139 (38.6)	157 (43.5)		271 (40.8)	25 (44.6)		107 (39.8)	189 (41.8)		257 (40.1)	39 (48.8)	

*SDH* succinate dehydrogenase.

*includes negative and low-positive cases.

### Clnicopathologic features according the phenotype of SDH expression in breast cancer

The SDHA/SDHB expression phenotypes in these 712 breast tumors were, in order of frequency, SDHA(+)/SDHB(+) > SDHA(-)/SDHB(–) > SDHA(-)/SDHB(+) > SDHA(+)/SDHB(–). The stroma phenotypes were SDHA(–)/SDHB(-) > SDHA(+)/SDHB(+) > SDHA(–)/SDHB(+) > SDHA(+)/SDHB(–) (Table 7).

**Table 7:** **Clinicopathologic features according to the SDH expression phenotype in breast cancer**

Factors	Tumor phenotype					Stroma phenotype				
	SDHA (+)/ SDHB	SDHA (+)/ SDHB	SDHA (−)/ SDHB	SDHA (-)/ SDHB	***P***	SDHA (+)/ SDHB	SDHA (+)/ SDHB	SDHA (−)/ SDHB	SDHA (−)/ SDHB	***P***
	(+) ***n*** = 679 (%)	(−) ***n*** = 1 (%)	(+) ***n*** = 18 (%)	(−) ***n*** = 23 (%)		(+) ***n*** = 42 (%)	(−) ***n*** = 14 (%)	(+) ***n*** = 38 (%)	(−) ***n*** = 627 (%)	
Histologic grade					0.038					0.150
I/II	450 (66.3)	1 (100.0)	13 (72.2)	20 (87.0)		25 (59.5)	7 (50.0)	26 (68.4)	426 (67.9)	
III	229 (33.7)	0 (0.0)	5 (27.8)	3 (13.0)		17 (40.5)	7 (50.0)	12 (31.6)	201 (32.1)	
Tumor stage					**0.495**					0.640
T1	329 (48.5)	0 (0.0)	6 (33.3)	15 (65.2)		20 (47.6)	7 (50.0)	24 (63.2)	299 (47.7)	
T2/T3	350 (51.5)	1 (100.0)	12 (66.7)	8 (34.8)		22 (52.4)	7 (50.0)	14 (36.8)	328 (52.3)	
Nodal stage					0.491					0.314
N0	398 (58.6)	1 (100.0)	11 (61.1)	15 (65.2)		21 (50.0)	10 (71.4)	20 (52.6)	374 (59.6)	
N1/N2/N3	281 (41.4)	0 (0.0)	7 (38.9)	8 (34.8)		21 (50.0)	4 (28.6)	18 (47.4)	253 (40.4)	
ER					0.284					0.205
Negative	248 (36.5)	1 (100.0)	7 (38.9)	5 (21.7)		16 (38.1)	10 (71.4)	14 (36.8)	221 (35.2)	
Positive	431 (63.5)	0 (0.0)	11 (61.1)	18 (78.3)		26 (61.9)	4 (28.6)	24 (63.2)	406 (64.8)	
PR					0.793					0.336
Negative	324 (47.7)	1 (100.0)	11 (61.1)	10 (43.5)		21 (50.0)	11 (78.6)	17 (44.7)	297 (47.4)	
Positive	355 (52.3)	0 (0.0)	7 (38.9)	13 (56.5)		21 (50.0)	3 (21.4)	21 (55.3)	330 (52.6)	
HER-2					**0.567**					**0.001**
Negative	539 (79.4)	0 (0.0)	14 (77.8)	20 (87.0)		26 (61.9)	13 (92.9)	21 (55.3)	513 (81.8)	
Positive	140 (20.6)	1 (100.0)	4 (22.2)	3 (13.0)		16 (38.1)	1 (7.1)	17 (44.7)	114 (18.2)	
Molecular type					0.057					**<0.001**
Luminal A	281 (41.4)	0 (0.0)	6 (33.3)	15 (65.2)		14 (33.3)	2 (14.3)	11 (28.9)	275 (43.9)	
Luminal B	160 (23.6)	0 (0.0)	5 (27.8)	3 (13.0)		13 (31.0)	2 (14.3)	13 (34.2)	140 (22.3)	
HER-2	63 (9.3)	1 (100.0)	3 (16.7)	2 (8.7)		9 (21.4)	1 (7.1)	9 (23.7)	50 (8.0)	
TNBC	175 (25.8)	0 (0.0)	4 (22.2)	3 (13.0)		6 (14.3)	9 (64.3)	5 (13.2)	162 (25.8)	
Ki-67 LI (%, mean ± SD)	17.5 ± 18.7	8.0 ± 0.0	16.8 ± 14.0	10.0 ± 10.2	0.260	17.8 ± 15.3	24.3 ± 22.8	22.1 ± 19.0	16.8 ± 18.4	0.161
Tumor recurrence	59 (8.7)	0 (0.0)	3 (16.7)	1 (4.3)	0.955	4 (9.5)	1 (7.1)	2 (5.3)	56 (8.9)	0.877
Patient death	56 (8.2)	0 (0.0)	3 (16.7)	1 (4.3)	0.968	3 (7.1)	1 (7.1)	3 (7.9)	53 (8.5)	0.727

*SDH* succinate dehydrogenase.

SDHA(+)/SDHB(+) tumors tended to have higher histologic grades than SDHA(+)/SDHB(–) and SDHA(–)/SDHB(-) tumors (*P* = 0.038). Stromal SDHA(–)/SDHB(+) tumors showed the highest frequency of HER-2 positivity, while stromal SDHA(+)/SDHB(-) tumors showed lowest HER-2 positivity (*P* = 0.001). Stromal SDHA/SDHB phenotype differed significantly between the molecular subtypes, SDHA(+)/SDHB(+) being most frequent in Luminal A and least in TNBC tumors. The SDHA(+)/SDHB(–) phenotype was most frequent in TNBC and least frequent in HER-2 subtype tumors; SDHA(–)/SDHB(+) was most frequent in Luminal B and least in TNBC; and SDHA(−)/SDHB(–) was most frequent in the Luminal A and least in the HER-2 subtype (*P* < 0.001).

### Prognostic significance of HIF-1α and SDH expression status

In univariate analysis, neither HIF-1α nor SDH expression was significantly related to patient outcome (Table 8). In multivariate Cox analysis (Table 9), factors negatively associated with DFS were T stage (Hazard ratio: 2.535, 95% CI: 1.376 − 4.670, *P* = 0.003), lymph node metastasis (Hazard ratio: 2.257, 95% CI: 1.335 − 3.816, *P* = 0.002), and high histologic grade (Hazard ratio: 1.685, 95% CI: 1.013 − 2.804, *P* = 0.045). Factors negatively associated with OS were T stage (Hazard ratio: 2.218, 95% CI: 1.229 − 4.001, *P* = 0.008), lymph node metastasis (Hazard ratio: 1.831, 95% CI: 1.076 − 3.115, *P* = 0.026), high tumor expression of SDHA (Hazard ratio: 4.157, 95% CI: 1.657 − 10.432, *P* = 0.002), and low SDHB expression (Hazard ratio: 3.223, 95% CI: 1.295 − 8.025, *P* = 0.012).

**Table 8:** **Univariate analysis of disease-free survival and overall survival according to breast-tumor expression of hypoxia-related proteins**

Parameters	Number of patients/ recurrence/death	Disease-free survival		Overall survival	
		Mean survival (95% CI) months	***P***-value	Mean survival(95% CI) months	***P***-value
Nuclear HIF-1α			0.911		0.943
Negative	697/61/58	126 (122 − 130)		129 (127 − 132)	
Positive	24/2/2	120 (108 − 133)		131 (122 − 141)	
Cytoplasmic HIF-1α			N/A		N/A
Negative	717/63	N/A		N/A	
Positive	4/0	N/A		N/A	
Tumoral SDHA			0.670		0.085
Negative/Low	360/35/26	125 (121 − 129)		131 (128 − 135)	
High	361/28/34	126 (120 − 132)		126 (121 − 130)	
Stromal SDHA			0.853		0.901
Negative	665/58/56	126 (123 − 130)		130 (127 − 132)	
Positive	56/5/4	115 (101 − 130)		121 (107 − 134)	
Tumoral SDHB			0.124		0.715
Negative/Low	269/31/26	123 (119 − 128)		129 (125 − 133)	
High	452/32/34	126 (121 − 131)		129 (125 − 132)	
Stromal SDHB			0.821		0.981
Negative	641/57/54	126 (122 − 130)		130 (127 − 132)	
Positive	80/6/6	119 (110 − 128)		123 (114 − 131)	

*SDH* succinate dehydrogenase P-value by log-rank test.

**Table 9:** **Multivariate analysis for breast-cancer survival**

Parameters	Disease-free survival			Overall survival		
	Hazard ratio	95% CI	***P***-value	Hazard ratio	95% CI	***P***-value
T stage			**0.003**			**0.008**
T1 versus T2-3	2.535	1.376 − 4.670		2.218	1.229 − 4.001	
N stage			**0.002**			**0.026**
N0 versus N1-3	2.257	1.335 − 3.816		1.831	1.076 − 3.115	
Histologic grade			**0.045**			0.372
I/II versus III	1.685	1.013 − 2.804		1.276	0.748 − 2.176	
Nuclear HIF-1α			0.599			0.486
Negative versus Positive	0.682	0.163 − 2.846		0.599	0.142 − 2.528	
Tumor SDHA			0.290			**0.002**
Negative/Low versus High	1.570	0.681 − 3.620		4.157	1.657 − 10.432	
Stromal SDHA			0.727			0.747
Negative versus Positive	1.254	0.352 − 4.468		0.807	0.218 − 2.978	
Tumor SDHB			0.096			**0.012**
Negative/Low versus High	0.498	0.219 − 1.131		3.223	1.295 − 8.025	
Stromal SDHB			0.712			0.965
Negative versus Positive	0.802	0.248 − 2.590		0.976	0.327 − 2.908	

*SDH* succinate dehydrogenase.

## Discussion

Germline defects in SDH, particularly in SDHA and SDHB, have been detected in several tumor types, including pheochromocytomas (Van Nederveen et al. 2009;Astuti et al. 2001), paragangliomas (Van Nederveen et al. 2009; Astuti et al. 2001; Baysal 2003;Burnichon et al. 2010), GISTs (Gaal et al. 2011;Gill et al. 2010b; Gill et al. 2011a), and renal cell carcinomas (Gill et al. 2011b). Until recently, the accurate detection of SDH mutation depended primarily on direct sequencing and western blotting, methods that a clinical laboratory may find too costly and time-consuming. However, in a study of paraganglioma and pheochromocytoma, Van Nederveen *et al.* demonstrated the high sensitivity of immunohistochemical methods to detect germline mutations in SDH (Van Nederveen et al. 2009). While the tumors with SDHB, SDHC, or SDHD germline mutations exhibited a loss of SDHB immmunoexpression with intact SDHA expression, tumors with SDHA germline mutations exhibited a loss of expression of both SDHA and SDHB.

Using similar methods in the present study, we found that 23 of 721 breast cancer patients (3.19%) had SDHA mutation (SDHA–/SDHB– expression) and one patient (0.1%) had an SDHB mutation (SHDA+/SDHB– expression; Table 7). As few previous studies have evaluated SDH mutation in breast cancer, these findings provide a starting point for future investigations. However, a previous study reported that SDH germline mutations or variants occur in some patients with Cowden syndrome (CS) who do not present the expected PTEN mutation. Compared with patients positive for germline PTEN mutation, these CS/CS-like individuals develop cancers of the breast, thyroid, and kidney at higher frequencies (Ni et al. 2008). Therefore, some breast cancer patients may be expected to have SDH mutations.

Findings in this study may be limited in that the sensitivity of immunohistochemistry in detecting SDH mutation as compared to direct sequencing has not been tested in breast cancer. We have assumed a degree of reliability in breast cancer similar to that of the detection of SDH germline mutations in paraganglioma and pheochromocytoma (Van Nederveen et al. 2009). To confirm SDH mutation, loss of SDH expression should be tested throughout the entire tumor (Barletta & Hornick 2012), whereas in this study, immunohistochemistry was performed on the tissue microarray only.

In previous studies of pheochromocytoma and GIST, SDH-deficient tumors showed complete loss of cytoplasmic granular expression (Miettinen et al. 2011; Gimenez-Roqueplo et al. 2003). Even though weak, focal or diffuse cytoplasmic staining was observed in a very few tumor cells of SDH-deficient tumor, this finding may be considered negative, because it is clearly distinguishable from the strong speckled expression pattern in surrounding non-neoplastic elements. However, stromal cells did not express SDHA and SDHB in most breast cancers in this study, which made it impossible to compare the staining intensity between tumor cells and internal normal controls (stromal cells). Therefore, expression in tumor cells was interpreted at three grade levels according to the proportion of cells stained: grade (1), negative (complete loss of expression); grade (2), low (expression in less than 50% of cells); and grade (3) high (expression in more than 50% of cells).

Several mechanisms have been proposed to explain the involvement of SDH mutation in tumorigenesis, among which a HIF-1α-pathway-dependent mechanism is the most famous. Loss-of-function mutation of SDH could result in an intracellular SDH accumulation, which in turn inhibits prolyl 4-hydroxylase (PHD), a negative regulator of HIF-1α (Cardaci & Ciriolo 2012; Barletta & Hornick 2012). The impaired PHD activity stabilizes HIF-1α under normoxic condition, which upregulates HIF target gene involved in cell growth stimulation and angiogenesis, thus contributing to tumor progression. As loss-of-function mutations in SDH are predicted to stabilize HIF-1α and upregulate HIF-1 transcriptional activity, we examined the expression of HIF-1α along with SDHA/SDHB. However, we found no close relationship between HIF-1α and SDH expression in these breast cancers.

SDH-deficient GISTs and renal cell carcinomas display characteristic histologic features distinguishable from tumors without SDH mutation (Miettinen et al. 2011; Gill et al. 2011b). Similarly, the SDHA+/SDHB- and SDHA-/SDHB- breast cancers in this study were distinguished by lower histologic grade (Table 7), and in the SDHB-negative tumors, by lower Ki-67 LI (Table 5). The luminal A breast cancer subtype showed the highest frequency of low/negative SDHA expression (*P* = 0.032, Table 3). Based on these results, SDH mutation in breast cancer is associated with low histologic grade and a less aggressive molecular subtype.

Patients with SDHA- and SDHB-negative breast cancers were also younger than patients with intact SDH expression, although in the case of SDHB, this difference was not statistically significant. In earlier studies of SDH mutations in renal cell carcinomas (Baysal 2003), GISTs (Miettinen et al. 2011), paragangliomas, and pheochromocytomas (Gimenez-Roqueplo et al. 2003), tumors also occurred at younger ages.

Metabolism in many malignant tumors is characterized by the Warburg effect, which is a high level of anaerobic glucose metabolism by glycolysis despite presence of oxygen with relatively low mitochondrial oxidative phosphorylation (OXPHOS) (Warburg 1956). In this study, more than 50% of breast cancers expressed SDHA and SDHB, which are key components of aerobic glucose metabolism through the TCA cycle and mitochondrial electron transport. This finding of high mitochondrial activity in breast cancer agrees with an earlier report that breast cancer cells show high expression of mitochondrial metabolic enzymes such as cytochrome C oxidase, NADH, and SDHB (Whitaker-Menezes et al. 2011).

Expression of Glut-1 and CAIX (indicators of glycolysis) has also been observed in breast cancers, most notably in high-grade tumors such as TNBC or basal-like carcinoma (Choi et al. 2013; Pinheiro et al. 2011). While breast cancers overall may express high levels of activity in both OXPHOS and glycolysis, the predominant mode of energy metabolism may differ according to the tumor type (Moreno-Sanchez et al. 2007: Kallinowski et al. 1989; Liu et al. 2001).

Although most breast cancers in this study were negative for SDH in stromal elements, stromal SDHB expression differed significantly among the molecular subtypes. The HER-2 subtype showed the highest and TNBC showed the lowest frequency of stromal SDHB expression (Table 3). However, in a previous study, stromal cells in breast cancer did not express mitochondrial metabolic enzymes (cytochrome c oxidase, NADH, or SDHB) (Whitaker-Menezes et al. 2011). Further studies will be required to determine the differences in stromal SDHB expression among breast cancer molecular subtypes.

In conclusion, loss of SDHA or SDHB expression was detected in approximately 3% of the breast cancers in this study. Patients with SDH-deficient breast cancers were younger at diagnosis and presented tumors of relatively low-grade histology.

### Consent

Written informed consent was obtained from the patient for the publication of this report and any accompanying images.

## Authors’ original submitted files for images

Below are the links to the authors’ original submitted files for images. Authors’ original file for figure 1 Authors’ original file for figure 2 Authors’ original file for figure 3
