# Supplementary figures and images for: Succinate dehydrogenase expression in breast cancer
Source: Springerplus. 2013 Jul 3;2(1):299. doi: 10.1186/2193-1801-2-299 (PMC3710570; doi:10.1186/2193-1801-2-299)

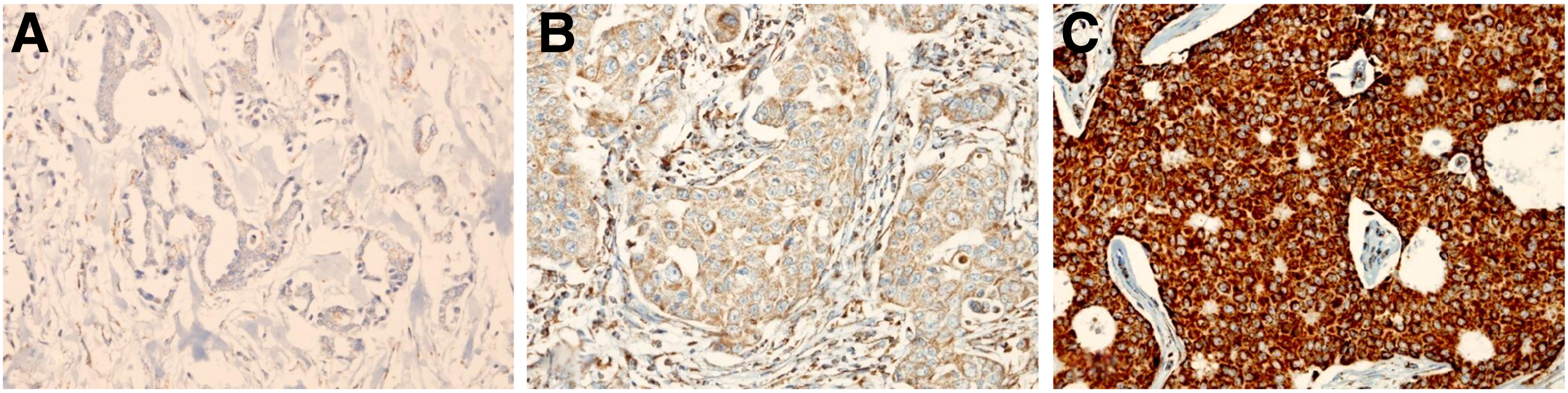

Supplement: Supplementary file 1 — Authors’ original file for figure 1 [file 40064_2013_378_MOESM1_ESM.tiff]

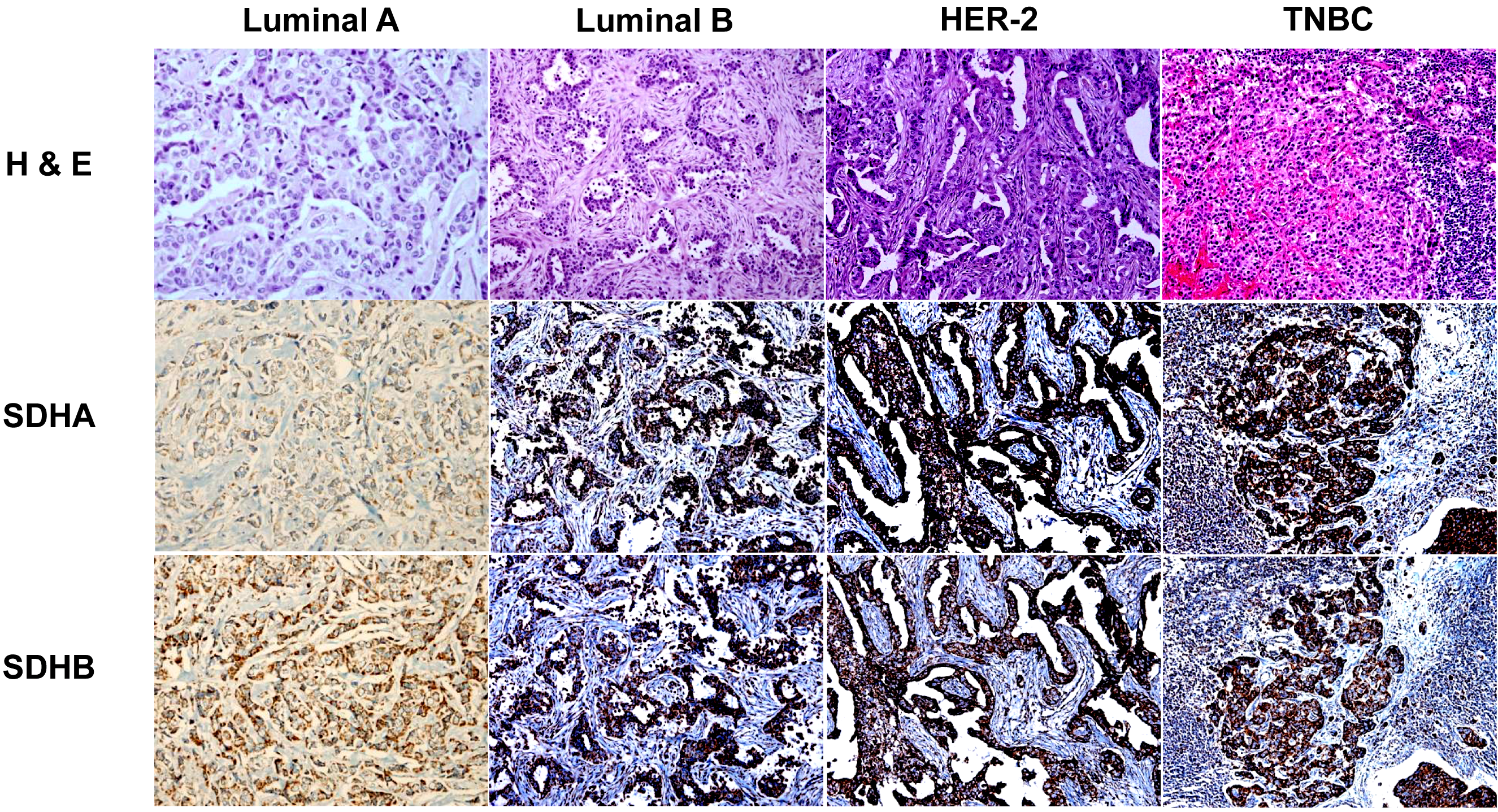

Supplement: Supplementary file 2 — Authors’ original file for figure 2 [file 40064_2013_378_MOESM2_ESM.png]

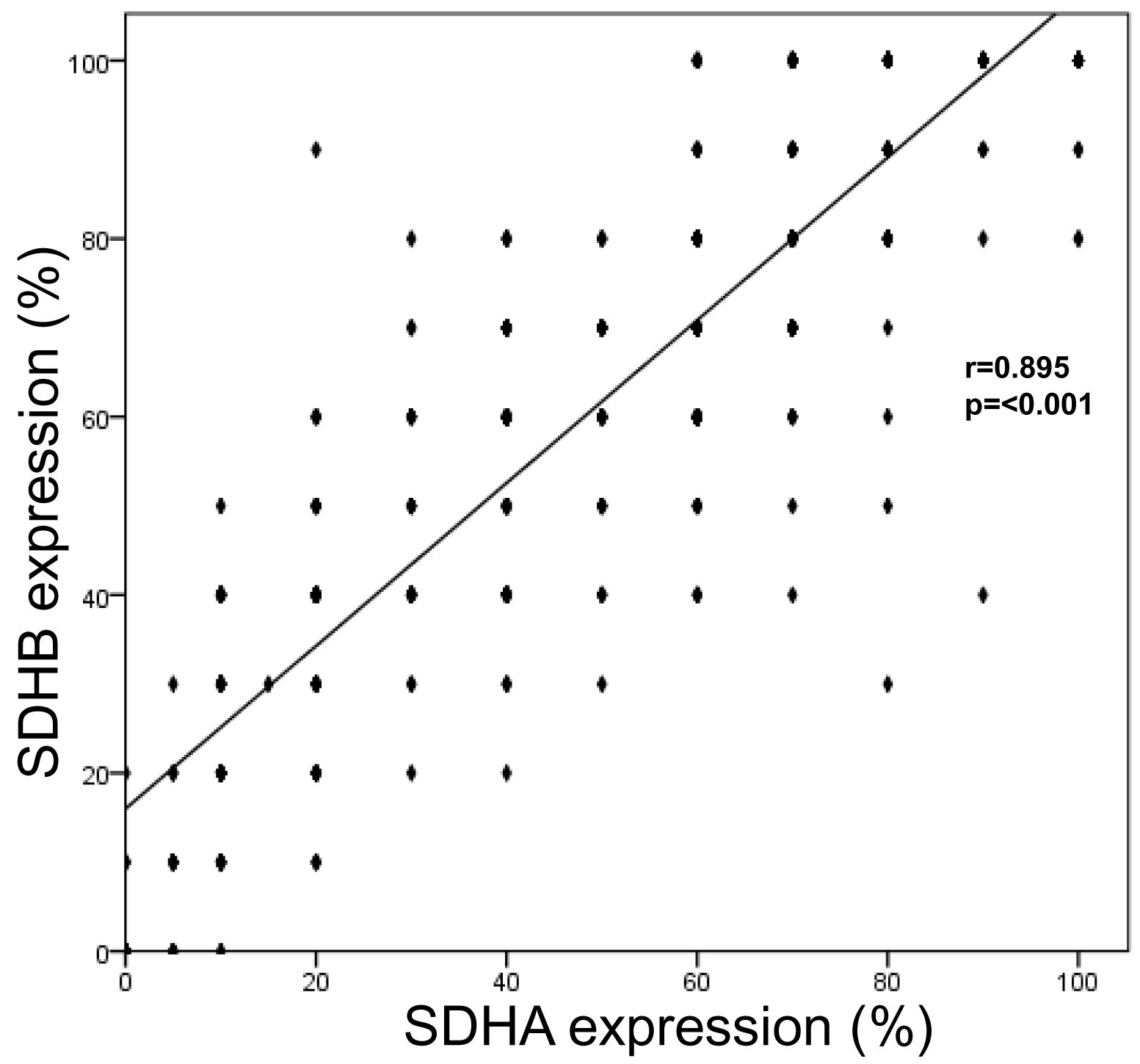

Supplement: Supplementary file 3 — Authors’ original file for figure 3 [file 40064_2013_378_MOESM3_ESM.png]
